# The relationship of topographical memory performance to regional neurodegeneration in Alzheimer's disease

**DOI:** 10.3389/fnagi.2012.00017

**Published:** 2012-07-04

**Authors:** George Pengas, Guy B. Williams, Julio Acosta-Cabronero, Tom W. J. Ash, Young T. Hong, David Izquierdo-Garcia, Tim D. Fryer, John R. Hodges, Peter J. Nestor

**Affiliations:** ^1^Department of Clinical Neurosciences, University of CambridgeCambridge, UK; ^2^Neuroscience Research Australia, SydneyNSW, Australia

**Keywords:** topographical memory, Alzheimer's, MRI, PET, multivariate, support vector, retrosplenial cortex

## Abstract

The network activated during normal route learning shares considerable homology with the network of degeneration in the earliest symptomatic stages of Alzheimer's disease (AD). This inspired the virtual route learning test (VRLT) in which patients learn routes in a virtual reality environment. This study investigated the neural basis of VRLT performance in AD to test whether impairment was underpinned by a network or by the widely held explanation of hippocampal degeneration. VRLT score in a mild AD cohort was regressed against gray matter (GM) density and diffusion tensor metrics of white matter (WM) (*n* = 30), and, cerebral glucose metabolism (*n* = 26), using a mass univariate approach. GM density and cerebral metabolism were then submitted to a multivariate analysis [support vector regression (SVR)] to examine whether there was a network associated with task performance. Univariate analyses of GM density, metabolism and WM axial diffusion converged on the vicinity of the retrosplenial/posterior cingulate cortex, isthmus and, possibly, hippocampal tail. The multivariate analysis revealed a significant, right hemisphere-predominant, network level correlation with cerebral metabolism; this comprised areas common to both activation in normal route learning and early degeneration in AD (retrosplenial and lateral parietal cortices). It also identified right medio-dorsal thalamus (part of the limbic-diencephalic hypometabolic network of early AD) and right caudate nucleus (activated during normal route learning). These results offer strong evidence that topographical memory impairment in AD relates to damage across a network, in turn offering complimentary lesion evidence to previous studies in healthy volunteers for the neural basis of topographical memory. The results also emphasize that structures beyond the mesial temporal lobe (MTL) contribute to memory impairment in AD—it is too simplistic to view memory impairment in AD as a synonym for hippocampal degeneration.

## Introduction

Memory impairment in Alzheimer's disease (AD) is often assumed to be a consequence of mesial temporal lobe (MTL) degeneration. The reasons for this are self-evident: neurofibrillary tangle (NFT) pathology in AD begins in the MTL (Braak and Braak, 1991); NFT load correlates with cognition in AD (Giannakopoulos et al., 2003); and the hippocampus is known to be atrophic in AD (Jack et al., 1997), including those at the mild cognitive impairment (MCI) stage whose deficit is restricted to memory (Pengas et al., 2010a). Such reasoning, however, might be an oversimplification. For instance, Braak and Braak (1991) used NFTs for their proposed staging protocol, precisely because they observed that NFT pathology followed a progressive pattern in AD; it is therefore expected that NFTs will correlate with other measures of disease severity. Furthermore, it is now well documented that prodromal AD is not simply a “hippocampal” disease: at a presymptomatic stage, carriers of familial, autosomal dominant AD mutations had both accelerated hippocampal and posterior cingulate volume loss (Scahill et al., 2002). This is also true of the MCI-stage of sporadic AD in which there is comparable atrophy of retrosplenial cortex, posterior cingulate cortex and hippocampus (Choo et al., 2010; Pengas et al., 2010a). Indeed, the earliest hypometabolic region identified in AD is the posterior cingulate/retrosplenial cortex (Minoshima et al., 1997; Nestor et al., 2003a) with posterior temporoparietal hypometabolism emerging as the next most affected isocortical region with disease progression (Nestor et al., 2003b; Chetelat et al., 2005).

This constellation of affected regions (MTL, posterior cingulate, retrosplenial cortex, posterior parietal lobe) is homologous with the network activated when healthy volunteers navigate in functional magnetic resonance imaging (MRI) paradigms (Ghaem et al., 1997; Maguire et al., 1998; Burgess et al., 2001; Ino et al., 2002; Shelton and Gabrieli, 2002; Rosenbaum et al., 2004; Wolbers et al., 2004; Wolbers and Buchel, 2005; Ekstrom and Bookheimer, 2007; Iaria et al., 2007; Ino et al., 2007). It was therefore, previously, hypothesized that topographical memory impairment would be sensitive and specific for early AD. The virtual route learning test (VRLT), in which subjects have to learn routes in a virtual reality environment, was devised and found to be highly sensitive and specific in distinguishing patients with early AD from controls. Moreover, when compared to a range of other memory tests, the VRLT most strongly correlated with real world navigation problems (Pengas et al., 2010b).

To our knowledge, only one previous study has investigated the neural basis of route learning impairment in AD but it did so as a two population voxel-based morphometry (VBM) contrast of gray matter density (GM) in subjects who got lost (*n* = 6 AD plus *n* = 5 MCI) versus those that did not (*n* = 4 AD plus *n* = 7 MCI plus *n* = 19 controls) who did not (deIpolyi et al., 2007). It reported no significant regions in a whole brain analysis but focusing on regions of interest in inferior parietal lobule, parahippocampal gyri and hippocampi, the authors reported atrophy in inferior parietal regions with a rightward bias as well as a tiny area in the right hippocampal tail. The use of regions of interest, the two population design, and inclusion of controls mean that only limited conclusions can be drawn from this study.

The aim of the present study, therefore, was to map the neural substrate for impaired route learning in an unbiased manner with multimodal imaging, using scores from the VRLT, in a cohort of mild AD patients. These behavioral data were derived from the earlier neuropsychological study (Pengas et al., 2010b) and, because the aim of the present work was to understand the basis of route learning impairment in AD, only patients with this condition (i.e., no controls) were included. VRLT scores were regressed with GM density (as a measure of regional brain atrophy), normalized ^18^F-fluorodeoxyglucose (FDG) radioactivity concentration—used as a measure of resting cerebral glucose metabolism corrected for inter-subject variation in basal metabolism—and diffusion tensor imaging (DTI) parameters of white matter tracts (WM). GM density and FDG data were also examined using a multivariate approach to specifically look at overall network contributions to navigation performance.

## Methods

### Subjects

A cohort of *n* = 30 patients with mild AD was identified for the study that had undergone detailed clinical and neuropsychological assessments of whom *n* = 16 patients had MCI (Petersen et al., 2001), and *n* = 14 met National Institute of Neurological and Communicable Diseases and Alzheimer's Disease and Related Disorders Association (NINCDS-ADRDA) criteria for probable AD (McKhann et al., 1984) at the time of scanning. The MCI subjects were followed up longitudinally after scanning to confirm progressive decline in all subjects, therefore indicating that their MCI status was due to probable AD. All 30 underwent the MRI protocol and a subset (*n* = 26 of which 14 were designated MCI and 12 AD when scanned) also had FDG—positron emission tomography (PET) imaging (demographics summarized in Table 1). These patients were derived from the earlier neuropsychological study of topographical memory in AD (Pengas et al., 2010b). The study was approved by the Local Research Ethics Committee and the Administration of Radioactive Substances Advisory Committee, UK. Written informed consent was obtained from all subjects and care-givers.

**Table 1:** **Demographics of the AD subjects that were included in the analyses (note: the PET subjects represent a subgroup of the MRI subjects; MMSE: Mini-mental state examination; ACE-r: Addenbrooke's Cognitive Examination-revised)**.

	**MRI subjects**	**PET subjects**
	**Mean ± SD (range)**	**Mean ± SD (range)**
N (sex)	30 (17*M*:13*F*)	26 (15*M*:11)
Age, yrs	69.2 ± 5.4 (59–78)	68.8 ± 5.6 (59–78)
Education, yrs	13.8 ± 3.0 (10–19)	13.8 ± 3.1 (10–19)
MMSE	24.6 ± 2.6 (18–28)	24.5 ± 2.6 (18–28)
ACE-r	74.2 ± 10.5 (55–88)	74.3 ± 10.2 (55–88)
VRLT error score	14.5 ± 5.3 (3–23)	14.5 ± 5.1 (3–23)

### Topographical memory assessment

Full details of the VRLT have been previously published (Pengas et al., 2010b). Briefly, the VRLT is s graded route learning task that employs four consecutively harder routes that subjects need to learn and reproduce by navigating with a joystick in a 3D first-person computer-generated virtual town. The task is scored in the number of errors a subject makes to complete all four routes, in a “learning-to-criterion” paradigm (Pengas et al., 2010b).

### Imaging

#### Image acquisition

***MRI.*** Within days of the behavioral data collection, MR images were acquired on a Siemens Trio 3T system (Siemens Medical Systems, Erlangen, Germany) equipped with gradient coils capable of 45 mT/m and a slew rate of 200T/m/s, and a 12-channel phased-array total imaging matrix head-coil (Siemens Medical Systems, Erlangen, Germany). At acquisition, the field of view was aligned to stereotactic space: the anterior commissure—posterior commissure line was aligned with the axial plane and the inter-hemispheric fissure was aligned along the sagittal plane. In addition, the scanning bed was adjusted to place the scanner isocentre at the thalamus in the mid-sagittal plane. Volumetric T1-weighted images were obtained using 3D magnetisation-prepared radio-frequency pulses and rapid gradient-echo (MP-RAGE) sampling (relaxation time (TR)/ echo time (TE)/ inversion time (TI)/ number of excitations (NEX) = 2300 ms/ 2.86 ms/ 900 ms/ 1; flip angle 9; matrix 192 × 192; 144 slices; voxel size 1.25 × 1.25 × 1.25 mm^3^ isotropic). Diffusion-weighted images (DWI) were acquired using a twice-refocused single-shot echo-planar imaging pulse sequence (Reese et al., 2003), with parameters TR/TE/NEX = 7800 ms/ 90 ms/ 1; matrix 96 × 96; 63 contiguous axial slices; isotropic voxel resolution of 2 × 2 × 2 mm^3^; bandwidth of 1628 Hz/pixel and echo spacing of 0.72 ms). The diffusion tensor was acquired with diffusion-sensitising gradient orientations along 63 non-collinear directions (*b* = 1000 s/mm^2^) that were maximally spread by considering the minimal energy arrangement of point charges on a sphere and one scan without diffusion weighting (*b* = 0 s/mm^2^, b0). Parallel acquisition of independently reconstructed images was allowed for, using generalised autocalibrating partially parallel acquisitions or GRAPPA (Griswold et al., 2002), with acceleration factor of 2 and 39 reference lines.

***FDG-PET.*** FDG-PET scans were acquired within a few weeks of the MRI scans for all patients, using a GE Advance scanner (GE Medical Systems, Milwaukee, WI, USA) in 3D mode (voxel size 2.34 × 2.34 × 4.25 mm^3^, field of view 30.0 × 30.0 × 15.3 cm^3^). Subjects were scanned after a 6 h fast in a dimly lit, quiet room, without using ear-plugs or blindfolds. A 150MBq FDG intravenous bolus injection was given over 30 s. Prior to the acquisition of emission images 35–55 min after injection (4 × 5 min), a 10 min geometrically windowed, coincidence mode transmission scan was performed using rotating germanium-68 rods for attenuation correction. PET emission images were reconstructed using the PROMIS 3D filtered back-projection algorithm (Kinnahan and Rogers, 1989) with corrections applied for dead time, randoms, normalisation, scatter, attenuation, sensitivity, and decay. Arterial sampling was not performed.

#### Data processing

***Voxel-based morphometry.*** Statistical parametric mapping 2005 (SPM5, http://fil.ion.ac.uk/spm) was employed to evaluate voxel-wise GM density using VBM (Ashburner and Friston, 2000). Recent studies have shown that skull-stripping and radio-frequency bias correcting MR images improve the performance of warping procedure so this was undertaken using a previously described algorithm (Acosta-Cabronero et al., 2008; Pereira et al., 2010). The resulting warped GM segments were modulated to compensate for volumetric differences introduced into the warped images, and smoothed using an 8 mm full-width half-maximum (FWHM) isotropic Gaussian kernel. Pre-processing and warping procedures need reasonable initial estimates; hence the origin of each structural volume was set manually to the anterior commissure prior to pre-processing. A relative masking threshold of 0.2 was applied for SPM5 analyses. The GM, WM and cerebrospinal fluid segments from SPM5 were summed together to calculate the total intracranial volume (Pengas et al., 2009), and entered as a nuisance covariate in the statistical regressions.

***FDG-PET.*** Mean FDG (35–55 min) radioactivity concentration maps were generated and their origins were also reset to the anterior commissure. The resulting volumes were skull-stripped using BET2 (*f* = 0.7, *g* = 0) (Smith, 2002), rigidly aligned to their corresponding pre-processed structural image and re-sliced (sinc interpolated) to the voxel size of the structural image using the VTK CISG registration toolkit v2.0.0 (Rueckert et al., 1999). Aligned mean FDG maps were then transformed into stereotactic space using the SPM5 warp transforms of pre-processed volumes and re-sampled to 2 mm isotropic using 7th degree b-spline interpolation. Finally, mean FDG maps were normalized by multiplying all voxels by the scaling factor required to equalize the mean cerebellar radioactivity concentration of each subject to the mean cerebellar radioactivity concentration of all subjects (Ichimiya et al., 1994), and smoothed with a 16 mm FWHM kernel. A relative masking threshold of 0.8 was applied for SPM5 analyses.

***Diffusion tensor imaging.*** The FSL package (http://www.fmrib.ox.ac.uk/fsl/) was employed to process and analyze the DWI data. First, each diffusion-weighted volume was affine-aligned to its corresponding b0 image using FMRIB's linear image registration tool v5.4.2 (Jenkinson and Smith, 2001) to correct for possible motion artefacts and eddy-current distortions. Prior to fitting the tensor, brain masks of each b0 image were generated using BET2 with *f* = 0.1 and *g* = 0. FMRIB's diffusion toolbox v2.0 was then used to fit the tensor and compute the orthogonal elements at each brain voxel, from which fractional anisotropy (FA), axial diffusivity (λ_1_), radial diffusivity (RD) and mean diffusivity (MD) metrics were derived. Spatial normalization was performed to a target image; this was the map that required the least amount of non-linear warping to match all other images, which was then affine-aligned into MNI152 standard space. The combination of the two transformations was applied to each subject's FA image, and all warped FA maps were then averaged to create the mean FA template, from which the mean FA skeleton is derived (FA > 0.2). Finally, all subjects' normalized FA, λ_1_, RD, and MD data were projected onto the skeleton for statistical analysis. The tract-based spatial statistics (TBSS) approach (Smith et al., 2006), whereby the nearest most relevant tract center in each subject's spatially normalized FA image is projected onto the mean FA skeleton containing the center of all tracts common to all subjects, was used to perform voxel-wise statistics at the tract centers only, thus minimizing the effect of residual misregistration.

#### Statistical analysis

***Univariate analyses.*** Whole brain analyses were carried out by regressing the VRLT error score with each of the three different imaging modalities: GM density, normalized FDG radioactivity concentration and diffusion metrics (λ_1_, RD, MD, and FA). GM density and normalized FDG radioactivity concentration were analyzed using voxel-based multiple linear regressions in SPM5 and illustrated at a statistical threshold of *p* (uncorrected) <0.005 and with no voxel extent threshold (*k* = 0). Diffusion analyses employed permutation-based non-parametric inference on unsmoothed statistical maps in TBSS; 10,000 permutations of the data were generated to test against using “randomise v2.1,” and cluster-like structures were enhanced using the threshold-free cluster enhancement algorithm (Smith and Nichols, 2009). Previous work has shown that λ_1_, MD, and RD can be more sensitive to diffusivity changes in AD than FA (Acosta-Cabronero et al., 2010). Therefore λ_1_, MD, RD (in addition to FA) were each assessed using multiple linear regression at *p* (uncorrected) <0.05. It should be highlighted, however, that there is presently uncertainty as to how changes in these various diffusion metrics relate to neuronal loss in WM; as such, the diffusion analyses should only be considered as exploratory at this time (See “Discussion” also).

***Multivariate analyses.*** Support vector machines for use in regression contexts [support vector regression (SVR)], allow for cognitive scores to be predicted using independently acquired data such as imaging, and therefore to interrogate the algorithm as to which brain areas influenced the algorithm the most, i.e., which are most relevant to the predicted cognitive score.

The general method of use for statistical learning tools has two phases. The first is a “supervised learning” phase, where the model is “trained” on a subset of the data and is optimized over a cost function, such that it outputs optimal results for that training set. A separate test on the remaining subset of the data then validates the generalizability of the learned model. As the present dataset was relatively small, the data cannot reasonably be split into learning and testing subsets (as small subsets inevitably lead to overfitting), and so cross-validation utilized “leave-one-out” tests instead. In this strategy, one subject's image is removed and the algorithm learns over the rest of the dataset. Then a prediction is made, based on the learned model, for the left-out image. This is then repeated over all subjects, and the accuracy is computed over all tests. The correlation between predicted scores and actual scores for each image then gives a measure of how well the technique performed, and how reliable the model it outputs is. Importantly, if this correlation was not statistically significant, further analyses (to identify relevant brain regions) were not performed.

In the case of regression, optimization takes place by ensuring that predictions over the training set have minimal error, whilst maintaining as robust a predictive function as possible. There are two adjustable parameters, C, the regularisation parameter, which restricts how much influence a single training example can have on the learned model, and ε, an upper bound for the maximum acceptable error allowed without penalty. The predictive form is:

$$Prediction=\sum_{i}^{SVs}\alpha_{i}k\left(x_{i},z\right)+b$$

where summation is over all training examples, α's are hyper-parameters giving the influence each training example has on the final model, *b* is a normalisation constant, and *k*(*x*_*i*_,*z*) is a kernel function, which allows a model to take a non-linear form.

Visualization of the model is simple in the case of a linear kernel. The functional form of the classifier can be simplified by using the concept of the weight vector, ***w***:

$$w=\sum_{i}^{SVs}\alpha_{i}x_{i}$$

The predictive form of the SVR technique is then:

Prediction=w⋅z+b

As the dot product of weight vector ***w*** with test vector ***z*** is taken, larger values of ***w*** represent regions of the trained model that are more sensitive to change in voxel intensity. In the present study, the SVR was implemented using SVM_light and employing default values from this algorithm for C and ε (Joachims, 1999). Displays of a thresholded version of the weight vector have been previously used in neuroscience work with support vector machines, e.g., Klöppel et al. (2008).

Interpretation of a weight vector is slightly different to interpretation of the more traditional mass univariate scheme. In a mass univariate scheme, an individual voxel is regressed to the score variable, and hence has an individual *p*-value associated with its correlation. In this multivariate case, a weighted version of the entire field of voxels is regressed to the score variable, and hence it is the overall pattern which has a *p*-value for correlation—the present technique cannot provide *p*-values for individual voxels, only for the entire pattern. Given that cognitive functions rely on distributed neural systems, this multivariate methodology is potentially very powerful as it offers the possibility to explain task performance at a network level.

## Results

### Univariate analyses

#### Atrophy (GM density) correlations

**Figure 1:**
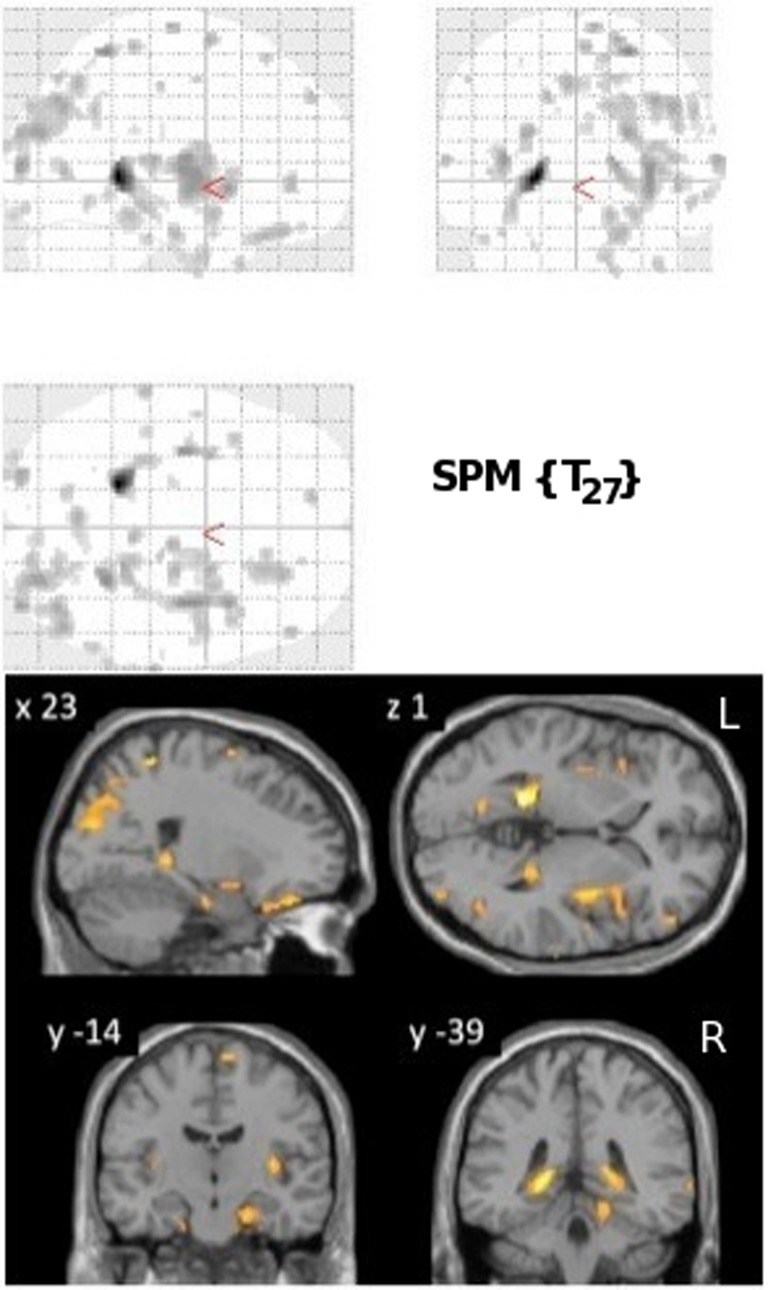
**Results of the univariate linear regression of VRLT error score with GM density.** *Above panel*: glass brain; *below panel*: selected slices projected onto a single subject template. The slice labeled “*y* = −39” best illustrates the bilateral isthmus/retrosplenial lesion.

VRLT performance correlated bilaterally with GM density in the region of the isthmus/retrospenial junction and extreme tail of the hippocampus. Patchy correlations were also evident in parahippocampal gyrus (PHG), insula, parietal and frontal cortices that were bilateral but with greater right hemisphere involvement (Figure 1).

#### Metabolism (FDG) correlations

**Figure 2:**
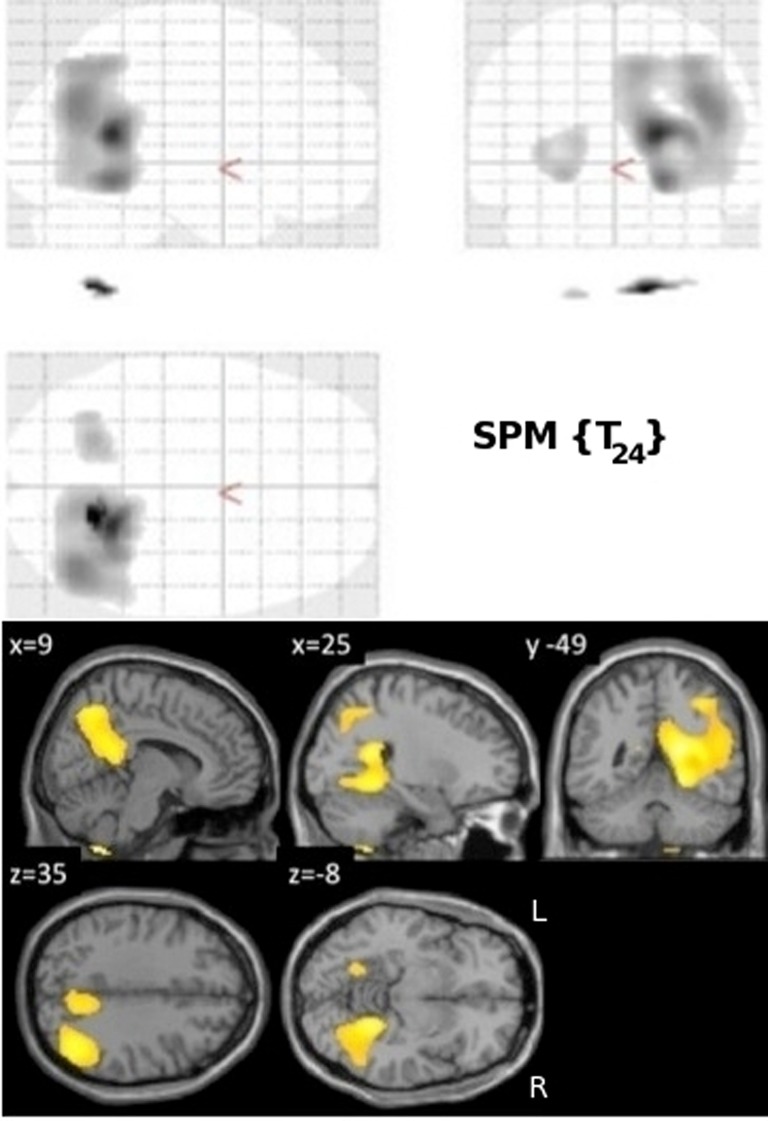
**Results of the univariate linear regression of VRLT error score with FDG metabolism.** *Above panel*: glass brain; *below panel*: selected slices projected onto a single subject template.

Normalized FDG radioactivity concentration correlation with VRLT performance identified a confluent region spanning right posterior cingulate, retrosplenial cortex, precuneus, lateral posterior parietal cortex, and posterior PHG. A less extensive and less significant cluster was seen on the left side involving posterior parietal cortex (Figure 2).

#### Diffusion (WM) correlations

**Figure 3:**
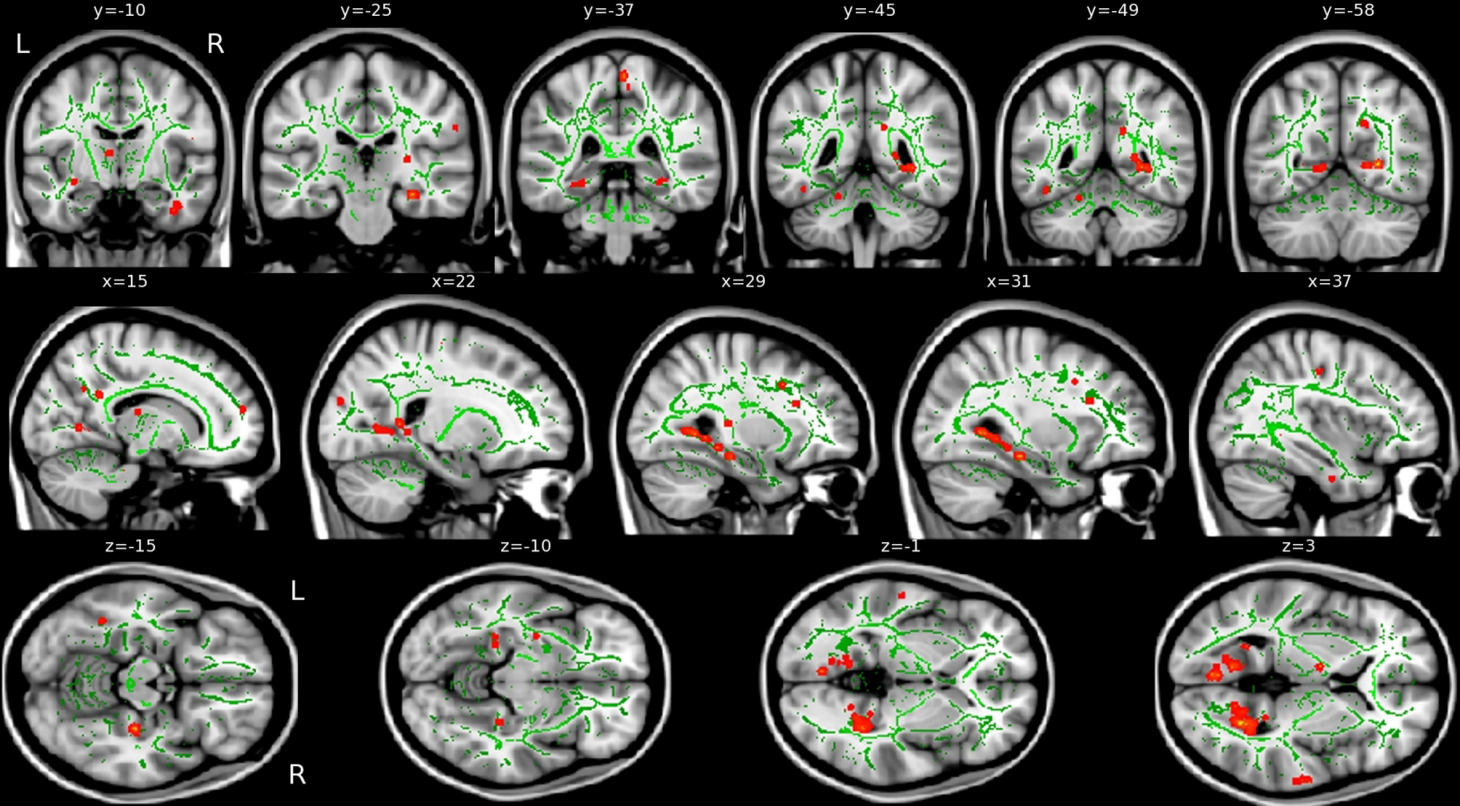
**Areas of correlation between increased axial diffusion (λ_1_, red) and VRLT error score.** The TBSS “skeleton” (i.e., the white matter tract centers on which the statistics are computed) is green.

VRLT performance correlated with λ_1_ bilaterally in the WM of the cingulum bundle in the posterior cingulate/retrosplenial region. On the right side this was more extensive and confluently extended rostrally in the PHG to the level of the hippocampal head in the *y*-plane (Figure 3). RD, MD, and FA failed to produce confluent results in any tract.

### Multivariate analyses

**Figure 4:**
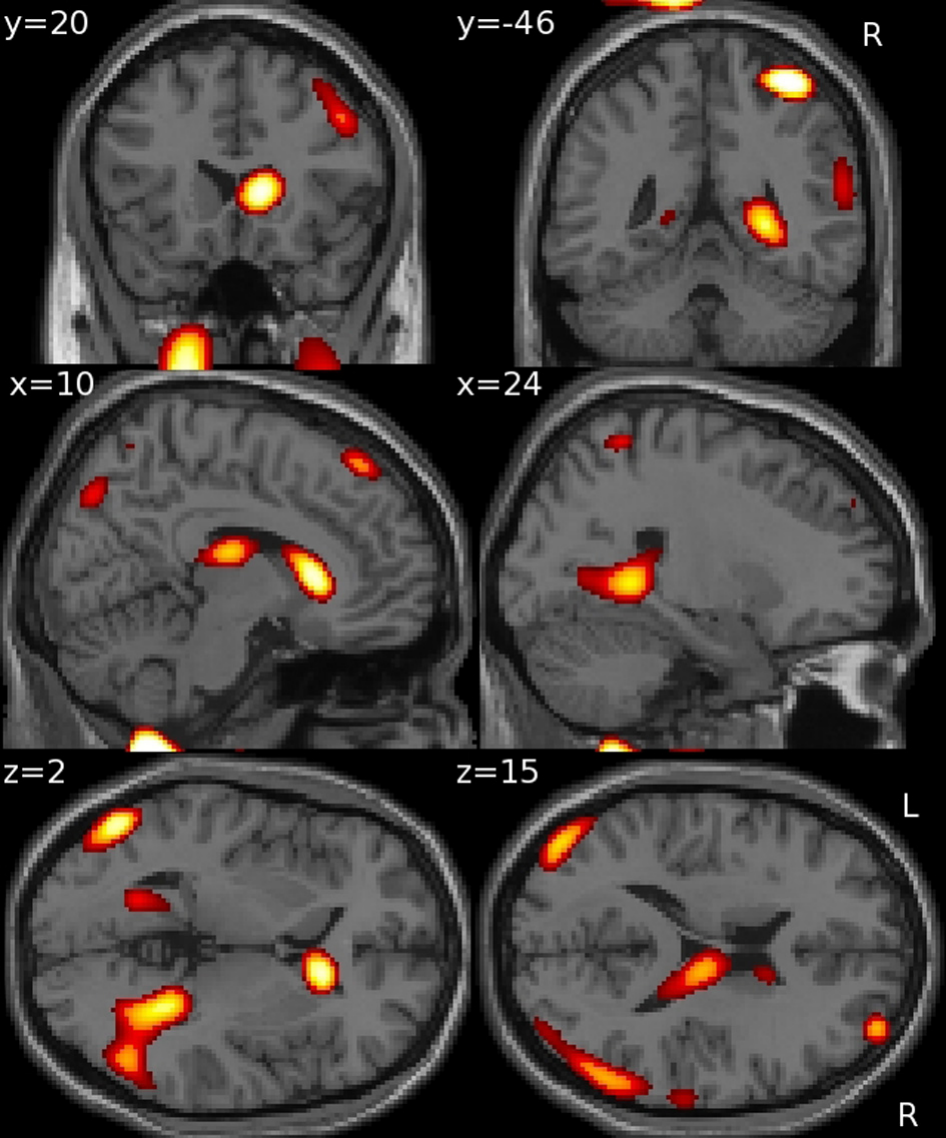
**Multivariate support vector regression of VRLT performance with normalized FDG metabolism**.

**Figure 5:**
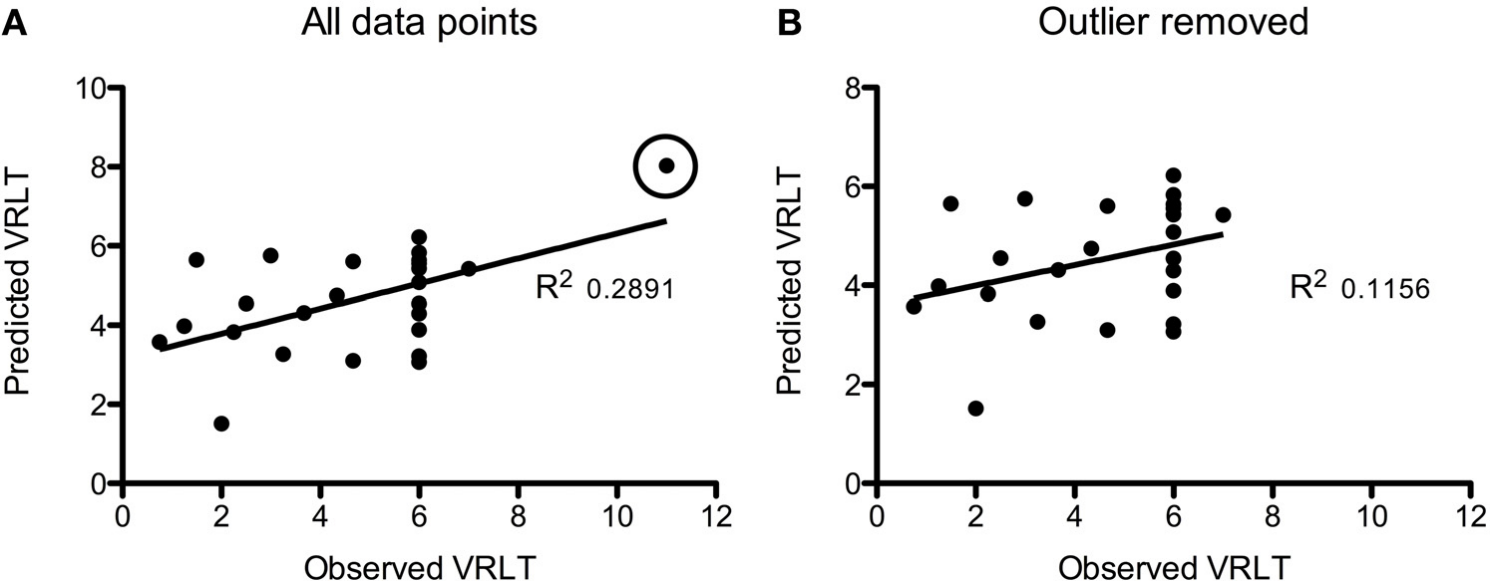
**Plots of the Support Vector Regression (predicted versus observed VRLT score). (A)** Shows the full cohort: note that one data point (circled) appeared to be a potential outlier. Removing this data point **(B)** diminished the strength of the correlation though it remained significant (*p* = 0.05).

Using GM density within an SVR model to predict VRLT performance did not produce values that correlated significantly with the true values (2-tailed *p* = 0.19). There was a significant predictive correlation between actual and predicted VRLT performance using normalized FDG radioactivity concentration (2-tailed *p* < 0.005). This involved bilateral postero-lateral parietal and retrosplenial cortices as well as right caudate nucleus, right medio-dorsal thalamus and a small area of right dorso-lateral prefrontal cortex. Overall, the regression was more extensive in the right hemisphere (Figure 4). Plotting the results of the SVR found that one data point was a potential outlier, re-running the correlation with this data point excluded diminished the significance of the correlation (1-tailed *p* = 0.05) (Figure 5).

### Convergence of results across methods and modalities

**Figure 6:**
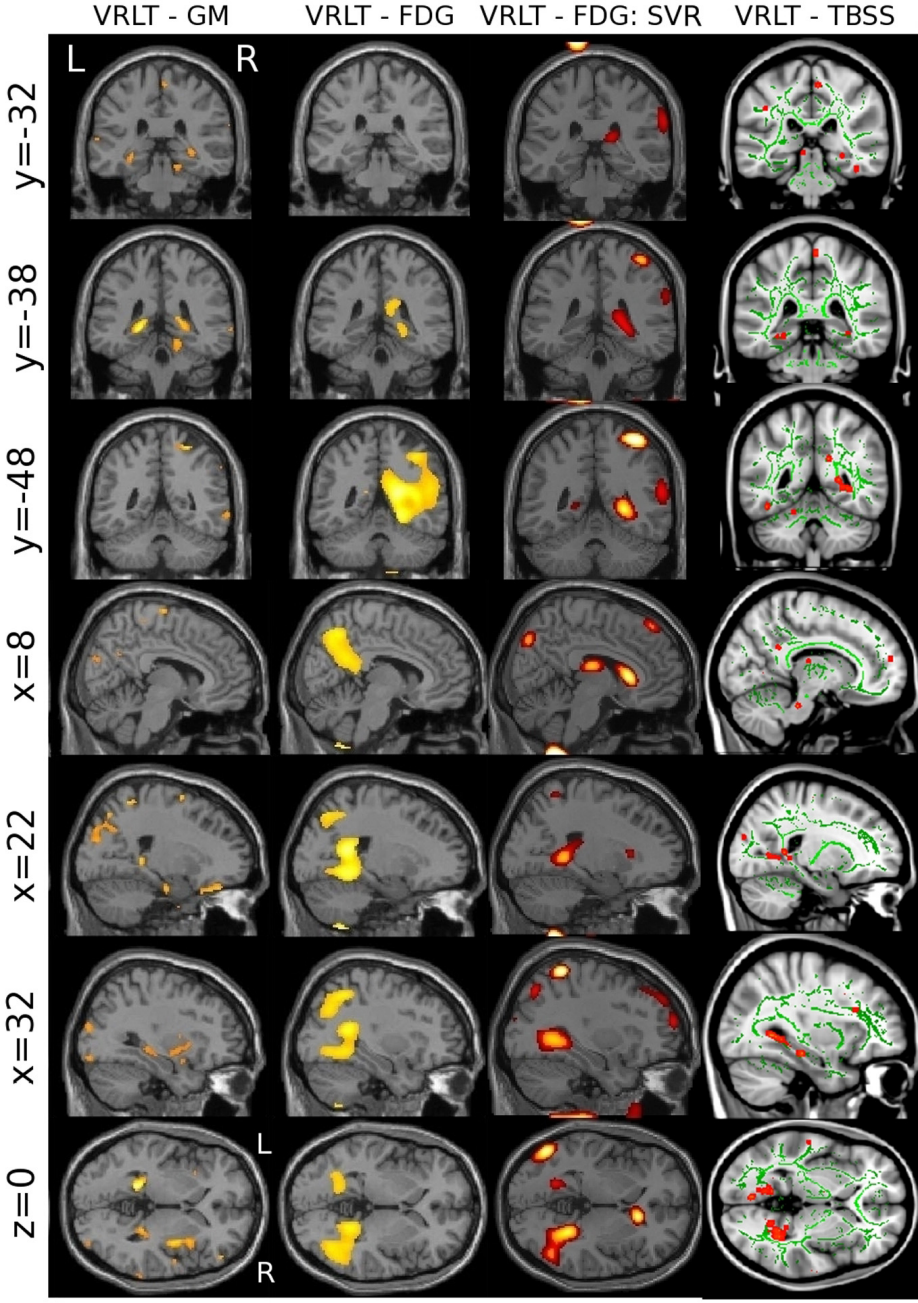
**Convergence of results across different imaging modalities and analysis techniques.** Note that the retrosplenial/isthmus correlation is common to all analyses (see rows *y* = −38 and *z* = 0).

The univariate analyses of GM and FDG and the multivariate FDG analysis identified a common area of correlation in the right retrospenial cortex/isthmus region extending to the region of the hippocampal tail. Axial diffusion (λ_1_) in WM adjacent to this region was also significantly correlated (Figure 6).

## Discussion

Performance on the VRLT, in which subjects attempt to learn routes in a virtual environment, was recently shown to have exquisite sensitivity for detecting impairment in very early AD; it also showed excellent ecological validity in that it correlated strongly with real world endorsements of route-finding difficulty (Pengas et al., 2010b). Univariate analyses of GM density, FDG (glucose metabolism) and WM axial diffusion found convergent correlations with the right retrosplenial/isthmus/posterior cingulate/hippocampal tail region. The multivariate analysis of FDG yielded a more distributed network of correlation that included this same region as well as lateral parietal association cortex, right caudate nucleus, and right dorso-medial thalamus. With the exception of the lateral parietal and retrosplenial regions, the network was exclusively right-sided. Though the present results offer considerably more detail, they are in general agreement with the GM density region of interest results of deIpolyi et al. (2007) who reported greater right side atrophy in AD and MCI patients who got lost, and involvement of the extreme right hippocampal tail (n.b. their region of interest analysis included hippocampi, parahippocampal gyri, and inferior parietal lobules).

Correlation of topographical memory impairment with the retrosplenial region is in agreement with behavioral data from focal lesions to this region (Takahashi et al., 1997; Maeshima et al., 2001). Similarly, functional neuroimaging studies of healthy subjects navigating in virtual environments activate the retrosplenial region (Ghaem et al., 1997; Maguire et al., 1998; Maguire, 2001; Ino et al., 2002; Wolbers et al., 2004). It also resonates with the observation that this area is the earliest detectable hypometabolic region in the MCI-stage of AD (Nestor et al., 2003b). The present findings suggest that this lesion is a significant contributor to the emergence of memory impairment in AD. Furthermore, the observation that both metabolism and GM density in this region correlated with VRLT performance suggests that this is not just a functional lesion but, rather, a direct consequence of local neurodegeneration. The correlation with axial diffusion in the WM subtending this region would also be consistent with local degeneration causing disruption to axonal projections from this area. As already mentioned, however, the diffusion analysis should only be treated as exploratory at this time; none of the other diffusion metrics correlated with VRLT performance, and, at present, too little is known about the dynamics of change between specific diffusion metrics and axonal loss to have strong expectations for how these variables may relate to each other.

The multivariate SVR analysis which estimates a significance level to the pattern of cerebral correlates as whole, and therefore could be viewed as giving a more informed picture of network level change, yielded a more complex picture than the mass univariate approach with respect to FDG. In addition to the retrosplenial lesion, this analysis revealed that lateral parietal regions—with a right-sided predominance—were also significantly related to VRLT performance. These findings highlight the homology between the fMRI pattern of activation during navigation (Ghaem et al., 1997; Maguire et al., 1998; Mellet et al., 2000; Burgess et al., 2001; Wolbers et al., 2004; Moffat et al., 2007) and the imaging profile in AD (Minoshima et al., 1997; Baron et al., 2001; Chetelat et al., 2002; Scahill et al., 2002; Nestor et al., 2003b; Acosta-Cabronero et al., 2010; Pengas et al., 2010a) that was the inspiration for developing this test. The analysis also highlighted right medio-dorsal thalamus in the correlation with VRLT performance. This location has not been a major feature of the spatial memory network in fMRI studies. Lesions to the thalamus are, however, an established cause of human amnesia (Stuss et al., 1988; Hodges and McCarthy, 1993) and, moreover, this area is part of the limbic-diencephalic network that is specifically hypometabolic in the amnesic prodrome of AD (Nestor et al., 2003a).

The failure to detect a significant network correlate with the multivariate GM SVR might suggest that the metabolic findings are predominantly physiological and separate from local degeneration (atrophy). It is important to emphasize, however, that this quite likely reflects the far greater sensitivity of FDG to detect local degeneration compared to GM density measurements. This phenomenon can be illustrated by contrasting the findings from two studies of the posterior cortical atrophy form of AD—one that examined glucose metabolism (Nestor et al., 2003c) and another that examined GM density (Whitwell et al., 2007). The lesion distribution in the two studies was identical suggesting a tight concordance between cerebral metabolism and atrophy; the key difference was that the FDG-PET result was generated from 16 subjects (*n* = 6 patients; *n* = 10 controls) whereas the GM density result came from 76 subjects (*n* = 38 in both patient and controls groups).

One finding in the multivariate FDG analysis probably does, however, represent a physiological network co-variance—this being the striking emergence that the right caudate nucleus was exerting an influence on performance. The right caudate nucleus is the one region identified to be part of the spatial memory network that is not a prominent component of the landscape of early degeneration in AD. It is, however, specifically activated during egocentric spatial memory tasks in healthy volunteers (Maguire et al., 1998; Doeller et al., 2008), and so would be expected to engage in a route learning test. Furthermore, there seems no reason to expect any disease-related caudate metabolism alterations in AD to be strongly right lateralized. We, therefore, propose that the unilateral right caudate finding in the current study offers direct evidence that this region is co-varying with posterior cortical regions involved in topographical memory performance. This observation reinforces the findings of previous fMRI studies in healthy volunteers that the right caudate nucleus is intimately involved in topographical memory processing using the complimentary model of a neurodegenerative disease that impairs this ability.

A very relevant negative in this study was the absence of prominent hippocampal correlation with task performance. Some analyses identified involvement of the extreme tail of the hippocampus, though, even then, it was never restricted to the hippocampus and could possibly have even been arising from correlation with neighbouring structures. Nevertheless, if real, it would be consistent with fMRI evidence that the tail may be the critical hippocampal component in spatial memory (Doeller et al., 2008). Arguably the more important point was that excluding the caudal couple of millimeters, no evidence was identified for hippocampal involvement in a topographical memory task that was previously shown to be highly sensitive to early AD. Without the current analyses, it is likely that many would assume topographical, indeed any kind of, memory test impairment in AD was a consequence of hippocampal damage. The present results challenge this view as an oversimplification. It would be overstating the case to suggest that the degree of hippocampal degeneration found in early AD is irrelevant to memory impairment—even in the present study it is possible that it exerted an influence that failed to reach statistical significance. It is, however, worth reflecting that the evidence that hippocampal degeneration causes memory impairment in AD is largely biased by the fact that studies finding correlation between these variables in the past have typically not examined regions beyond the MTL. Although many such studies found significant correlations (Kohler et al., 1998; de Toledo-Morrell et al., 2000a,b; Grundman et al., 2003; Reitz et al., 2009; Stoub et al., 2010), given that both MTL degeneration and memory impairment are a function of disease severity, one could ask, how could they fail to correlate? This risk of non-causal correlation is exemplified in studies in which non-mnemonic functions such as naming (Wilson et al., 1996) also correlated with MTL degeneration. The present results also offer explanation for the observation that patients with the non-Alzheimer disorder, semantic dementia, can perform the VRLT normally (Pengas et al., 2010b) yet have been previously shown to have greater MTL degeneration than is seen in AD (Chan et al., 2001; Galton et al., 2001; Nestor et al., 2006).

In conclusion, this study sought to investigate the neural basis of topographical memory impairment in AD. The traditional mass univariate regression approach employed with several different imaging modalities found correlations that particularly converged on a region encompassing retrosplenial/isthmus/posterior cingulate cortex and possibly hippocampal tail. A relatively novel multivariate approach, however, revealed that a, predominantly right hemispheric, network that reflected elements of (1) the limbic-diencephalic network known to be abnormal in incipient AD and (2) the topographical network highlighted in past fMRI studies of healthy volunteers, underpinned task performance in AD. These results, therefore, offer lesion evidence to corroborate observations made in healthy subjects regarding human route learning. Moreover, the findings highlight that memory impairment in AD is network-driven and unlikely to be simply a consequence of MTL damage.

### Conflict of interest statement

The authors declare that the research was conducted in the absence of any commercial or financial relationships that could be construed as a potential conflict of interest.

## References

[B1] Acosta-Cabronero J. Williams G. B. Pengas G. Nestor P. J. (2010). Absolute diffusivities define the landscape of white matter degeneration in Alzheimer's disease. Brain 133(Pt 2), 529–539 10.1093/brain/awp257 19914928

[B2] Acosta-Cabronero J. Williams G. B. Pereira J. M. Pengas G. Nestor P. J. (2008). The impact of skull-stripping and radio-frequency bias correction on grey-matter segmentation for voxel-based morphometry. Neuroimage 39, 1654–1665 10.1016/j.neuroimage.2007.10.051 18065243

[B3] Ashburner J. Friston K. J. (2000). Voxel-based morphometry-the methods. Neuroimage 11(6 Pt 1), 805–821 10.1016/j.neuroimage.2008.01.003 10860804

[B4] Baron J. C. Chetelat G. Desgranges B. Perchey G. Landeau B. de la Sayette V. Eustache F. (2001). *In vivo* mapping of gray matter loss with voxel-based morphometry in mild Alzheimer's disease. Neuroimage 14, 298–309 10.1006/nimg.2001.0848 11467904

[B5] Braak H. Braak E. (1991). Neuropathological stageing of Alzheimer-related changes. Acta Neuropathol. 82, 239–259 1759558 10.1007/BF00308809

[B6] Burgess N. Maguire E. A. Spiers H. J. O'Keefe J. (2001). A temporoparietal and prefrontal network for retrieving the spatial context of lifelike events. Neuroimage 14, 439–453 10.1016/j.neuroimage.2005.05.057 11467917

[B7] Chan D. Fox N. C. Scahill R. I. Crum W. R. Whitwell J. L. Leschziner G. Rossor A. M. Stevens J. M. Cipolotti L. Rossor M. N. (2001). Patterns of temporal lobe atrophy in semantic dementia and Alzheimer's disease. Ann. Neurol. 49, 433–442 11310620

[B8] Chetelat G. Desgranges B. De La Sayette V. Viader F. Eustache F. Baron J. C. (2002). Mapping gray matter loss with voxel-based morphometry in mild cognitive impairment. Neuroreport 13, 1939–1943 12395096 10.1097/00001756-200210280-00022

[B9] Chetelat G. Eustache F. Viader F. De La Sayette V. Pelerin A. Mezenge F. Hannequin D. Dupuy B. Baron J. C. Desgranges B. (2005). FDG-PET measurement is more accurate than neuropsychological assessments to predict global cognitive deterioration in patients with mild cognitive impairment. Neurocase 11, 14–25 10.1080/13554790490896938 15804920

[B10] Choo I. H. Lee D. Y. Oh J. S. Lee J. S. Lee D. S. Song I. C. Youn J. C. Kim S. G. Kim K. W. Jhoo J. H. Woo J. I. (2010). Posterior cingulate cortex atrophy and regional cingulum disruption in mild cognitive impairment and Alzheimer's disease. Neurobiol. Aging 31, 772–779 10.1016/j.neurobiolaging.2008.06.015 18687503

[B11] deIpolyi A. R. Rankin K. P. Mucke L. Miller B. L. Gorno-Tempini M. L. (2007). Spatial cognition and the human navigation network in AD and MCI. Neurology 69, 986–997 10.1212/01.wnl.0000271376.19515.c6 17785667

[B12] de Toledo-Morrell L. Dickerson B. Sullivan M. P. Spanovic C. Wilson R. Bennett D. A. (2000a). Hemispheric differences in hippocampal volume predict verbal and spatial memory performance in patients with Alzheimer's disease. Hippocampus 10, 136–142 10.1002/(SICI)1098-1063(2000)10:2<136::AID-HIPO2>3.0.CO;2-J 10791835

[B13] de Toledo-Morrell L. Goncharova I. Dickerson B. Wilson R. S. Bennett D. A. (2000b). From healthy aging to early Alzheimer's disease: *in vivo* detection of entorhinal cortex atrophy. Ann. N.Y. Acad. Sci. 911, 240–253 10.1111/j.1749-6632.2000.tb06730.x 10911878

[B14] Doeller C. F. King J. A. Burgess N. (2008). Parallel striatal and hippocampal systems for landmarks and boundaries in spatial memory. Proc. Natl. Acad. Sci. U.S.A. 105, 5915–5920 10.1073/pnas.0801489105 18408152 PMC2311337

[B15] Ekstrom A. D. Bookheimer S. Y. (2007). Spatial and temporal episodic memory retrieval recruit dissociable functional networks in the human brain. Learn. Mem. 14, 645–654 10.1101/lm.575107 17893237 PMC2044556

[B16] Galton C. J. Patterson K. Graham K. Lambon-Ralph M. A. Williams G. Antoun N. Sahakian B. J. Hodges J. R. (2001). Differing patterns of temporal atrophy in Alzheimer's disease and semantic dementia. Neurology 57, 216–225 11468305 10.1212/wnl.57.2.216

[B17] Ghaem O. Mellet E. Crivello F. Tzourio N. Mazoyer B. Berthoz A. Denis M. (1997). Mental navigation along memorized routes activates the hippocampus, precuneus, and insula. Neuroreport 8, 739–744 9106758 10.1097/00001756-199702100-00032

[B18] Giannakopoulos P. Herrmann F. R. Bussiere T. Bouras C. Kovari E. Perl D. P. Morrison J. H. Gold G. Hof P. R. (2003). Tangle and neuron numbers, but not amyloid load, predict cognitive status in Alzheimer's disease. Neurology 60, 1495–1500 12743238 10.1212/01.wnl.0000063311.58879.01

[B19] Griswold M. A. Jakob P. M. Heidemann R. M. Nittka M. Jellus V. Wang J. Kiefer B. Haase A. (2002). Generalized autocalibrating partially parallel acquisitions (GRAPPA). Magn. Reson. Med. 47, 1202–1210 10.1002/mrm.10171 12111967

[B20] Grundman M. Jack C. R. Jr. Petersen R. C. Kim H. T. Taylor C. Datvian M. Weiner M. F. DeCarli C. DeKosky S. T. van Dyck C. Darvesh S. Yaffe K. Kaye J. Ferris S. H. Thomas R. G. Thal L. J. (2003). Hippocampal volume is associated with memory but not monmemory cognitive performance in patients with mild cognitive impairment. J. Mol. Neurosci. 20, 241–248 14501003 10.1385/jmn:20:3:241

[B21] Hodges J. R. McCarthy R. A. (1993). Autobiographical amnesia resulting from bilateral paramedian thalamic infarction. A case study in cognitive neurobiology. Brain 116(Pt 4), 921–940 10.1093/brain/116.4.921 8353716

[B22] Iaria G. Chen J. K. Guariglia C. Ptito A. Petrides M. (2007). Retrosplenial and hippocampal brain regions in human navigation: complementary functional contributions to the formation and use of cognitive maps. Eur. J. Neurosci. 25, 890–899 10.1111/j.1460-9568.2007.05371.x 17298595

[B23] Ichimiya A. Herholz K. Mielke R. Kessler J. Slansky I. Heiss W. D. (1994). Difference of regional cerebral metabolic pattern between presenile and senile dementia of the Alzheimer type: a factor analytic study. J. Neurol. Sci. 123, 11–17 8064302 10.1016/0022-510x(94)90197-x

[B24] Ino T. Doi T. Hirose S. Kimura T. Ito J. Fukuyama H. (2007). Directional disorientation following left retrosplenial hemorrhage: a case report with fMRI studies. Cortex 43, 248–254 17405670 10.1016/s0010-9452(08)70479-9

[B25] Ino T. Inoue Y. Kage M. Hirose S. Kimura T. Fukuyama H. (2002). Mental navigation in humans is processed in the anterior bank of the parieto-occipital sulcus. Neurosci. Lett. 322, 182–186 10.1016/S0304-3940(02)00019-8 11897168

[B26] Jack C. R. Jr. Petersen R. C. Xu Y. C. Waring S. C. O'Brien P. C. Tangalos E. G. Smith G. E. Ivnik R. J. Kokmen E. (1997). Medial temporal atrophy on MRI in normal aging and very mild Alzheimer's disease. Neurology 49, 786–794 9305341 10.1212/wnl.49.3.786 PMC2730601

[B27] Jenkinson M. Smith S. (2001). A global optimisation method for robust affine registration of brain images. Med. Image Anal. 5, 143–156 10.1016/S1361-8415(01)00036-6 11516708

[B28] Joachims T. (1999). “Making large-scale SVM learning practical,” in Advances in kernel methods - Support vector learning, eds Schollkopf B. Burges C. Smola A. (Cambridge, MA: MIT press), 169–184

[B29] Kinnahan P. E. Rogers J. G. (1989). Analytic 3D image reconstruction using all detected events. IEEE Trans. Nucl. Sci. 36, 964–968

[B30] Klöppel S. Stonnington C. M. Chu C. Draganski B. Scahill R. I. Rohrer J. D. Fox N. C. Jack C. R. Jr. Ashburner J. Frackowiak R. S. (2008). Automatic classification of MR scans in Alzheimer's disease. Brain 131(Pt 3), 681–689 10.1093/brain/awm319 18202106 PMC2579744

[B31] Kohler S. Black S. E. Sinden M. Szekely C. Kidron D. Parker J. L. Foster J. K. Moscovitch M. Winocour G. Szalai J. P. Bronskill M. J. (1998). Memory impairments associated with hippocampal versus parahippocampal-gyrus atrophy: an MR volumetry study in Alzheimer's disease. Neuropsychologia 36, 901–914 10.1016/S0028-3932(98)00017-7 9740363

[B32] Maeshima S. Ozaki F. Masuo O. Yamaga H. Okita R. Moriwaki H. (2001). Memory impairment and spatial disorientation following a left retrosplenial lesion. J. Clin. Neurosci. 8, 450–451 10.1054/jocn.2000.0801 11535016

[B33] Maguire E. A. (2001). The retrosplenial contribution to human navigation: a review of lesion and neuroimaging findings. Scand. J. Psychol. 42, 225–238 10.1111/1467-9450.00233 11501737

[B34] Maguire E. A. Burgess N. Donnett J. G. Frackowiak R. S. Frith C. D. O'Keefe J. (1998). Knowing where and getting there: a human navigation network. Science 280, 921–924 10.1126/science.280.5365.921 9572740

[B35] McKhann G. Drachman D. Folstein M. Katzman R. Price D. Stadlan E. M. (1984). Clinical diagnosis of Alzheimer's disease: report of the NINCDS-ADRDA Work Group under the auspices of Department of Health and Human Services Task Force on Alzheimer's disease. Neurology 34, 939–944 6610841 10.1212/wnl.34.7.939

[B36] Mellet E. Briscogne S. Tzourio-Mazoyer N. Ghaem O. Petit L. Zago L. Etard O. Berthoz A. Mazoyer B. Denis M. (2000). Neural correlates of topographic mental exploration: the impact of route versus survey perspective learning. Neuroimage 12, 588–600 10.1006/nimg.2000.0648 11034866

[B37] Minoshima S. Giordani B. Berent S. Frey K. A. Foster N. L. Kuhl D. E. (1997). Metabolic reduction in the posterior cingulate cortex in very early Alzheimer's disease. Ann. Neurol. 42, 85–94 10.1002/ana.410420114 9225689

[B38] Moffat S. D. Kennedy K. M. Rodrigue K. M. Raz N. (2007). Extrahippocampal contributions to age differences in human spatial navigation. Cereb. Cortex 17, 1274–1282 10.1093/cercor/bhl036 16857855

[B39] Nestor P. J. Fryer T. D. Smielewski P. Hodges J. R. (2003a). Limbic hypometabolism in Alzheimer's disease and mild cognitive impairment. Ann. Neurol. 54, 343–351 10.1002/ana.10669 12953266

[B40] Nestor P. J. Fryer T. D. Ikeda M. Hodges J. R. (2003b). Retrosplenial cortex (BA 29/30) hypometabolism in mild cognitive impairment (prodromal Alzheimer's disease). Eur. J. Neurosci. 18, 2663–2667 10.1046/j.1460-9568.2003.02999.x 14622168

[B41] Nestor P. J. Caine D. Fryer T. D. Clarke J. Hodges J. R. (2003c). The topography of metabolic deficits in posterior cortical atrophy (the visual variant of Alzheimer's disease) with FDG-PET. J. Neurol. Neurosurg. Psychiatry 74, 1521–1529 10.1136/jnnp.74.11.1521 14617709 PMC1738241

[B42] Nestor P. J. Fryer T. D. Hodges J. R. (2006). Declarative memory impairments in Alzheimer's disease and semantic dementia. Neuroimage 30, 1010–1020 10.1016/j.neuroimage.2005.10.008 16300967

[B43] Pengas G. Hodges J. R. Watson P. Nestor P. J. (2010a). Focal posterior cingulate atrophy in incipient Alzheimer's disease. Neurobiol. Aging 31, 25–33 10.1016/j.neurobiolaging.2008.03.014 18455838

[B44] Pengas G. Patterson K. Arnold R. J. Bird C. M. Burgess N. Nestor P. J. (2010b). Lost and found: bespoke memory testing for Alzheimer's disease and semantic dementia. J. Alzheimers Dis. 21, 1347–1365 10.3233/JAD-2010-100654 21504124

[B45] Pengas G. Pereira J. M. Williams G. B. Nestor P. J. (2009). Comparative reliability of total intracranial volume estimation methods and the influence of atrophy in a longitudinal semantic dementia cohort. J. Neuroimaging 19, 37–46 10.1111/j.1552-6569.2008.00246.x 18494772

[B46] Pereira J. M. Xiong L. Acosta-Cabronero J. Pengas G. Williams G. B. Nestor P. J. (2010). Registration accuracy for VBM studies varies according to region and degenerative disease grouping. Neuroimage 49, 2205–2215 10.1016/j.neuroimage.2009.10.068 19892022

[B47] Petersen R. C. Stevens J. C. Ganguli M. Tangalos E. G. Cummings J. L. DeKosky S. T. (2001). Practice parameter: early detection of dementia: mild cognitive impairment (an evidence-based review). Report of the quality standards subcommittee of the American academy of neurology. Neurology 56, 1133–1142 10.1212/WNL.56.9.1133 11342677

[B48] Reese T. G. Heid O. Weisskoff R. M. Wedeen V. J. (2003). Reduction of eddy-current-induced distortion in diffusion MRI using a twice-refocused spin echo. Magn. Reson. Med. 49, 177–182 10.1002/mrm.10308 12509835

[B49] Reitz C. Honig L. Vonsattel J. P. Tang M. X. Mayeux R. (2009). Memory performance is related to amyloid and tau pathology in the hippocampus. J. Neurol. Neurosurg. Psychiatry 80, 715–721 10.1136/jnnp.2008.154146 19258354 PMC2785022

[B50] Rosenbaum R. S. Ziegler M. Winocur G. Grady C. L. Moscovitch M. (2004). “I have often walked down this street before”: fMRI studies on the hippocampus and other structures during mental navigation of an old environment. Hippocampus 14, 826–835 10.1002/hipo.10218 15382253

[B51] Rueckert D. Sonoda L. I. Hayes C. Hill D. L. Leach M. O. Hawkes D. J. (1999). Nonrigid registration using free-form deformations: application to breast MR images. IEEE Trans. Med. Imaging 18, 712–721 10.1109/42.796284 10534053

[B52] Scahill R. I. Schott J. M. Stevens J. M. Rossor M. N. Fox N. C. (2002). Mapping the evolution of regional atrophy in Alzheimer's disease: unbiased analysis of fluid-registered serial MRI. Proc. Natl. Acad. Sci. U.S.A. 99, 4703–4707 10.1073/pnas.052587399 11930016 PMC123711

[B53] Shelton A. L. Gabrieli J. D. (2002). Neural correlates of encoding space from route and survey perspectives. J. Neurosci. 22, 2711–2717 11923436 10.1523/JNEUROSCI.22-07-02711.2002 PMC6758311

[B54] Smith S. M. (2002). Fast robust automated brain extraction. Hum. Brain Mapp. 17, 143–155 10.1016/j.neuroimage.2008.10.066 12391568 PMC6871816

[B55] Smith S. M. Nichols T. E. (2009). Threshold-free cluster enhancement: addressing problems of smoothing, threshold dependence and localisation in cluster inference. Neuroimage 44, 83–98 10.1016/j.neuroimage.2008.03.061 18501637

[B56] Smith S. M. Jenkinson M. Johansen-Berg H. Rueckert D. Nichols T. E. Mackay C. E. Watkins K. E. Ciccarelli O. Cader M. Z. Matthews P. M. Behrens T. E. (2006). Tract-based spatial statistics: voxelwise analysis of multi-subject diffusion data. Neuroimage 31, 1487–1505 10.1016/j.neuroimage.2006.02.024 16624579

[B57] Stoub T. R. Rogalski E. J. Leurgans S. Bennett D. A. deToledo-Morrell L. (2010). Rate of entorhinal and hippocampal atrophy in incipient and mild AD: relation to memory function. Neurobiol. Aging 31, 1089–1098 10.1016/j.neurobiolaging.2008.08.003 18809228 PMC2873053

[B58] Stuss D. T. Guberman A. Nelson R. Larochelle S. (1988). The neuropsychology of paramedian thalamic infarction. Brain Cogn. 8, 348–378 3214590 10.1016/0278-2626(88)90059-0

[B59] Takahashi N. Kawamura M. Shiota J. Kasahata N. Hirayama K. (1997). Pure topographic disorientation due to right retrosplenial lesion. Neurology 49, 464–469 9270578 10.1212/wnl.49.2.464

[B60] Whitwell J. L. Jack C. R. Jr. Kantarci K. Weigand S. D. Boeve B. F. Knopman D. S. Drubach D. A. Tang-Wai D. F. Petersen R. C. Josephs K. A. (2007). Imaging correlates of posterior cortical atrophy. Neurobiol. Aging 28, 1051–1061 10.1016/j.neurobiolaging.2006.05.026 16797786 PMC2734142

[B61] Wilson R. S. Sullivan M. deToledo-Morrell L. Stebbins G. T. Bennett D. A. (1996). Association of memory and cognition in Alzheimer's disease with volumetric estimates of temporal lobe structures. Neuropsychology 10, 459–463

[B62] Wolbers T. Buchel C. (2005). Dissociable retrosplenial and hippocampal contributions to successful formation of survey representations. J. Neurosci. 25, 3333–3340 10.1523/JNEUROSCI.4705-04.2005 15800188 PMC6724902

[B63] Wolbers T. Weiller C. Buchel C. (2004). Neural foundations of emerging route knowledge in complex spatial environments. Brain Res. Cogn. Brain Res. 21, 401–411 10.1016/j.cogbrainres.2004.06.013 15511655

